# Psychological interventions improve quality of life despite persistent pain in endometriosis: results of a 3-armed randomized controlled trial

**DOI:** 10.1007/s11136-023-03346-9

**Published:** 2023-02-17

**Authors:** K. E. Hansen, B. Brandsborg, U. S. Kesmodel, A. Forman, M. Kold, R. Pristed, O. Donchulyesko, D. Hartwell, L. Vase

**Affiliations:** 1 Department of Public Health, Aarhus University, Bartholins Allé 2, 8000 Aarhus, Denmark; 2 Department of Psychology and Behavioral Sciences, School of Business and Social Sciences, Aarhus University, 8000 Aarhus, Denmark; 3 Department of Clinical Medicine, Aarhus University, 8210 Aarhus, Denmark; 4 Department of Obstetrics and Gynaecology, Aalborg University Hospital, 9000 Aalborg, Denmark; 5 Department of Clinical Medicine, Aalborg University, 9000 Aalborg, Denmark; 6 Department of Obstetrics and Gynaecology, Aarhus University Hospital, 8210 Aarhus, Denmark; 7 Department of Communication and Psychology, The Faculty of Social Sciences and Humanities (SSH), Aalborg University, 9000 Aalborg, Denmark; 8 Department of Psychological Health, Agder University, Agder, Norway; 9 Department of Obstetrics and Gynaecology, North Denmark Regional Hospital, Hjørring, Denmark; 10 Department of Gynaecology, Copenhagen University Hospital, Rigshospitalet, Copenhagen, Denmark

**Keywords:** Endometriosis, Quality of life, Chronic pelvic pain, Psychotherapy, Control condition, Mindfulness, Acceptance and commitment therapy

## Abstract

**Purpose:**

Despite standard medical treatment endometriosis is often associated with disabling pain and poor quality of life (QoL). Studies indicate that psychological interventions (PIs) may improve pain and QoL, yet studies on the effects of PIs for women with endometriosis are sparse and limited by low-quality study designs. Therefore, this study aimed, in a rigorous three-armed design, to evaluate the effect of PIs on chronic pelvic pain (CPP) and QoL in women with endometriosis.

**Methods:**

This three-armed parallel, multi-center randomized controlled trial included fifty-eight endometriosis patients reporting severe CPP [≥ 5 for pain intensity measured on a 0–10-point numeric rating scale (NRS)]. Patients were randomly assigned to (1) Specific mindfulness- and acceptance-based psychological intervention (MY-ENDO), (2) Carefully matched non-specific psychological intervention (Non-specific), or (3) A wait-list control group (WL). The primary outcome was pelvic pain intensity/unpleasantness measured on NRS. Secondary outcomes included endometriosis-related quality of life, workability, pain acceptance, and endometriosis-related symptoms. Differences in outcomes between groups at post-treatment follow-up were analyzed using mixed linear models. Analyses were performed on an intention-to-treat basis.

**Results:**

Compared to WL, psychological intervention (MY-ENDO + Non-specific) did not significantly reduce pain. However, psychological intervention did significantly improve the QoL-subscales ‘control and powerlessness’, ‘emotional well-being’, and ‘social support’ as well as the endometriosis-related symptoms ‘dyschezia’ and ‘constipation’. MY-ENDO was not superior to Non-specific.

**Conclusions:**

Women with endometriosis may have significant and large effects of psychological intervention on QoL despite an ongoing experience of severe CPP.

**Trial registration:**

12 April 2016, clinicaltrials.gov (NCT02761382), retrospectively registered.

## Plain English summary

Endometriosis is a chronic gynecological disease affecting 5–10% of women worldwide. It can lead to disabling pelvic pain and poor quality of life. The traditional treatments for painful endometriosis consist of medical and/or surgical treatment. However, these treatments are, in many cases, insufficient in relieving the pain and improving the quality of life of these women. This study aimed to examine whether a psychological treatment can improve pain and quality of life in women suffering from painful endometriosis. In addition, the study examines whether *mindfulness- and acceptance-based psychological intervention* is a more effective treatment than a *non-specific psychological intervention*.

The study demonstrated that psychological intervention does not lead to pain reduction in women with endometriosis. However, it significantly improved the quality of life of these women despite an ongoing experience of severe chronic pelvic pain. It also improved the endometriosis-related symptoms “constipation” and “pain during defecation”. Therefore, the study indicates that psychological intervention may be an appropriate strategy to manage symptoms and improve the quality of life in women with endometriosis, but a definitive decision on the preferred psychological modality (Mindfulness- and acceptance-based psychological intervention as compared to Non-specific psychological intervention) cannot be made. More research is needed before we can conclude whether one specific psychological intervention is to be preferred to best manage symptoms and improve the quality of life in women suffering from painful endometriosis.

## Background

Endometriosis is a chronic and often painful gynecological disease defined as the presence and growth of endometrium-like tissue outside the uterus, usually in the pelvis, where it causes bleeding, inflammation, and adhesions [1]. The estimated prevalence is 5–10% among women of reproductive age [1, 2]. Long-term symptoms include cyclical and chronic pelvic pain (CPP), dyspareunia, irritable bowel syndrome (IBS), infertility, and fatigue [3–5]. Endometriosis is associated with reduced psychological and social well-being [6–10], and its negative impact on all domains of quality of life (QoL) is well-documented. Thematic analysis has identified several key QoL domains in the areas of physical, psychological, and social health such as: (a) diagnostic delay and uncertainty, (b) everyday activities, (c) intimate relationships, (d) planning for and having children, (e) education and work, (f) medical- and self-management, and (g) mental health and emotional well-being [6, 8]. In addition, symptoms such as depression, anxiety, and perceived stress are frequent [11–13]. Studies indicate that women suffering from endometriosis-related pelvic pain display significantly lower QoL than women with asymptomatic endometriosis and healthy pain-free controls. Therefore, the negative impact on mental health and QoL seems to be associated with the number and severity of pain symptoms and not by having the diagnosis per se [14, 15].

Current standard treatment for painful endometriosis includes hormonal treatment, pain medication, and/or surgical resection of endometrial lesions. Despite such treatment, recurrence and development of chronic pain problems are frequent [16–21]. As psychological factors are likely to be important in modifying pain perception, psychological interventions (PIs) may be effective for pain reduction [22, 23]. Until now, empirical investigations of PIs for endometriosis have been sparse and limited by low-quality of the study designs including small pilot studies or insufficient control conditions that do not allow for a separation of the specific versus the non-specific effects [24, 25]. A small observational pilot study showed significant long-term effects of a mindfulness-based PI on endometriosis-related QoL [26, 27], but since the quality of control conditions is found to be associated with outcomes [22, 28], well-designed and rigorous randomized controlled trials (RCTs) on the effects of PIs on CPP and QoL in endometriosis are needed. Preferably, studies should include direct and validated pain measures such as a Numeric Rating Scale (NRS) in the assessment of endometriosis-related pain [29] and add clinically relevant experimental pain testing to tap into the pain modulatory system and investigate potential pain mechanisms [30].

Consequently, we conducted a three-armed RCT to test the effects of (1) a specific PI (MY-ENDO), (2) a matched non-specific PI (Non-specific) and (3) a waitlist control (WL) on CPP and QoL in women with endometriosis. The hypothesis was to find statistically significant improvements in CPP and a number of secondary outcomes for (1) PI (MY-ENDO + Non-specific) compared to WL and for (2) MY-ENDO compared to Non-specific.

## Methods

### Study design

Patients were randomly assigned to one of three conditions: (1) A specific mindfulness- and acceptance-based PI called “Mind Your ENDOmetriosis” (MY-ENDO), (2) A non-specific PI (Non-specific) that matched MY-ENDO in non-specific factors such as empathy, the therapeutic alliance, a cogent rationale, and expectations of improvement, but did not include the assumed specific ingredient, mindfulness or (3) A WL that involved treatment, as usual, to control for the natural fluctuations in pain [31]. Participants in the waitlist group were offered one of the two PIs after the end of the study period. All groups received medical treatment as usual. This design enabled a rigorous examination of the efficacy of MY-ENDO to clarify to which extent specific mindfulness- and acceptance ingredients are essential for the potential effects of this intervention.

The study was preregistered with The Danish Data Protection Agency (journal no. 2015-57-0002), approved by The Central Denmark Region Committees on Health Research Ethics (registration no. 1-10-72-138-15), and retrospectively registered at clinicaltrials.gov (NCT02761382). Data was collected from March 2016 to October 2018.

### Participants

Participants were recruited from three specialized outpatient clinics for endometriosis in Denmark and from the Danish Endometriosis Patients Association. All patients considered for inclusion underwent screening to assess in- and exclusion criteria. Inclusion criteria were: (a) 18–47 years old, (b) surgery or MRI-confirmed endometriosis diagnosis, (c) moderate to severe CPP (i.e., an average of ≥ 5 measured on an 11-point Numerical Rating Scale (NRS) from 0 = no pain to 10 = worst pain imaginable), (d) relevant clinical and surgical treatment according to the European Society of Human Reproduction and Embryology (ESHRE) guidelines for endometriosis [32] had been tried, (e) willingness to spend 30–45 min on homework 5–7 days a week for 10 weeks. Exclusion criteria were (a) other serious physical pain diseases (e.g., fibromyalgia, Crohn’s disease, Colitis Ulcerosa), (b) severe psychiatric diagnosis, (c) pregnancy or planned pregnancy during the study period, and (d) an estimated lack of mental or physical surplus to enter into a psychological treatment or linguistic or cultural barriers.

### Procedure

A letter was sent to interested patients with study details and a pain diary to be filled out before the screening session. At the screening, patients were informed about study requirements and screened for in- and exclusion criteria. They provided written informed consent before enrolment in the study and randomization. Patients were informed that they would be randomized to one of two different psychological interventions or a waitlist control group. This should keep participants blinded to the psychological method and intervention content in the comparison group. To keep the research group blinded to intervention assignment throughout data collection, a research assistant, not part of the research group, provided patients with an anonymous id-number used for data collection. The numbers were randomized in blocks of six by another research assistant using a computer-generated randomization list.

**Fig. 1:**
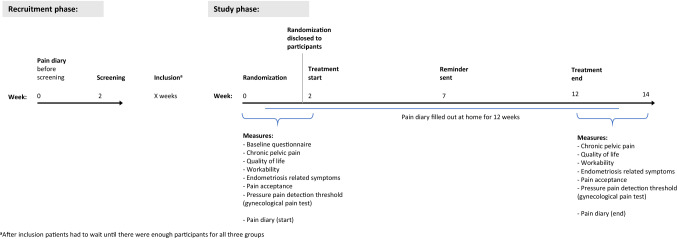
Study timeline

Questionnaires were sent to participants by postal mail, filled out, and returned. Baseline measurements were obtained during the 2 weeks period prior to treatment start, and post-intervention measurements were obtained during the 2 weeks post-treatment period. At home, patients also completed a 12-week pain diary starting 1 week pre-intervention until 1-week post-intervention. To investigate potential changes in pain processing and sensibility a female doctor carried out a gynecological experimental pain assessment during the 2-week period prior to treatment start and again during the 2 weeks post-treatment. However, the experimental pain assessment was optional and not required for participation (See Fig. 1. Study timeline).

### Interventions

#### MY-ENDO

MY-ENDO has been developed specifically for endometriosis. It is based on the manualized 8-week program called mindfulness-based stress reduction (MBSR) [33, 34] in combination with acceptance and commitment therapy (ACT) [35] but adjusted to better suit the specific needs and challenges of women with endometriosis. The specific details of the intervention are presented in Table 1. MY-ENDO consists of a manualized 10-week program including 3-h weekly group sessions with patient education about themes related to endometriosis, group therapy focusing on patients´ experiences and coping mechanisms in relation to these themes, and a variety of mindfulness and yoga exercises practiced during treatment sessions. Furthermore, participants were given a set of handouts (Table 1) and encouraged to practice 30–45 min of mindfulness meditation and yoga at home five to seven days a week.

**Table 1:** The detailed content of the Mindfulness- and Acceptance-based Psychological Intervention (MY-ENDO) and the Non-specific psychological intervention (Non-specific)

Session	Shared content		MY-ENDO (Mind Your ENDOmetriosis)		Non-specific	
	Patient education	Group therapy	Mindfulness- and acceptance exercises^a^	Yoga practices	Mindfulness control	Yoga control
1	Endometriosis	My history and experiences with endometriosis	Long body scan	–	Relaxation while listening to music	–
2	Chronic pelvic pain and pain mechanisms	My experiences with chronic pelvic pain and pain management	Long body scan, breathing meditation	Savasana, Chakravakasana, Pada Hastasana, Balasana, Jathara Parivartanasana	Relaxation while listening to music	Training exercises
3	Psychological and social impact of endometriosis	How does endometriosis affect my life today?	Short body scan, breathing meditation	Savasana, Chakravakasana, Adho mukha svanasana, Pada Hastasana, Ashwa-sanchalanasana, Balasana, Jathara Parivartanasana	Relaxation while listening to music	Training exercises
4	Stress and anxiety	My experiences with stress and anxiety	Defusion meditation, schemas about stress and anxiety, short body scan	Savasana, Supta Baddha Konasana, Pavanamuktasana, Janushirshasana, Upavishta Konasana, Ardha Matsyendrasana, Jathara Parivartanasana	Relaxation while listening to music	Training exercises
5	Depression and grief	My experiences with depression and grief	Defusion meditation, schemas on depressive feelings and grief, short body scan	Pranamasana, Hasta Uttanasana, Pada Hastasana, Ashwa-sanchalanasana, Adho mukha svanasana, Asthanga Namaskar, Bhujangasana	Relaxation while listening to music	Training exercises
6	Health, diet, and exercise	My experiences with diet and exercise. What is good health?	Mindful walking, mindfulness in everyday life (eating, bathing, etc.), and guided meditation “from thinking to sensing”	Hasta Uttanasana, Pada Hastasana, Uttanasana 2, Neck rolling, Virabhadrasana 2, Utthita trikonasana, Parivrtta trikonasana, Vrksasana	Relaxation while listening to music	Training exercises
7	Relations, sexuality, and fertility	How does endometriosis affect social relations, partnership, sexuality, family, and children?	Loving-kindness meditation, heart meditation	Supta Baddha Konasana, Setu Bandhasana, Chakravakasana, Pada Hastasana, Balasana	Relaxation while listening to music	Training exercises
8	Identity and meaning	How does endometriosis affect my identity and what is the meaning of my life?	Identifying values, “the values compass”, heart meditation	Supta Baddha Konasana, Setu Bandhasana, Jathara Parivartanasana, Neck rolling	Relaxation while listening to music	Training exercises
9	A good life with endometriosis	What is a good life with endometriosis to me?	Planning and accomplishing committed action, mountain meditation	Pranamasana, Hasta Uttanasana, Pada Hastasana, Ashwa-sanchalanasana, Adho mukha svanasana, Asthanga Namaskar, Bhujangasana	Relaxation while listening to music	Training exercises
10	Completion and looking forward: Repetition and how to move on from now		Participants choose meditation	Participants choose yoga	Relaxation while listening to music	Training exercises
Handouts	Endometriosis booklet		Mindfulness treatment booklet, USB with guided meditations, and 20 yoga cards with pictures and instructions		Non-specific treatment booklet, CD with relaxing music, and 20 training cards with pictures and instructions	

^a^During mindfulness training, patients were instructed to focus their attention on the target of observation (e.g., the body, breathing, or walking) as it was experienced in the present moment. When thoughts, feelings, or sensations arose, they were simply to be observed non-judgmentally, without any attempts to change them. When participants noticed that their minds had drifted away from the intended target in the present moment (e.g., their minds had drifted to memories, fantasies, or future events), they were asked to briefly acknowledge the attentional drift—without judgment—and then asked to return their attention to the present moment

#### Non-specific

To properly test whether MY-ENDO was truly superior to other psychological interventions and if the effects were due to specific mindfulness ingredients, the control condition had to be an intervention based on psychological principles. This means: (1) to have a cogent and acceptable rationale (2) to include corresponding therapeutic actions, and (3) to be delivered by trained therapists in a healing context with expectations that the therapy would be beneficial [36–38]. Therefore, the Non-specific intervention was developed by removing all aspects specific to MBSR and ACT from the MY-ENDO manual, while aspects related to more non-specific factors of psychological intervention were held constant (Table 1). (Data covering the details on the rationale, development, and influence of the Non-specific control are not included in this manuscript.) All guided mindfulness meditation and yoga were removed from the Non-specific treatment manual, but to control for (a) the time used on mindfulness meditation, (b) an auditory input (guiding), and (c) relaxation (often a result of mindfulness training) a detailed control for these specific elements was developed and added to the Non-specific intervention (see Table 1). The detailed control included relaxation while listening to soft and relaxing music and guided physical training (warm-up, muscle training, and stretching) intended for women with chronic pelvic pain. Participants were encouraged to practice 30–45 min of relaxation and physical training at home five to seven days a week. Also, the handouts were matched in detail (layout etc.) (Table 1).

#### Waitlist

The Wait-list group received medical treatment as usual and completed the same questionnaires and gynecological pain assessment as the intervention groups.

### Therapists

Two private practicing clinical psychologists, both licensed by the Danish board of psychologists and closely matched on essential parameters (training, apprenticeship, and competence), were recruited to deliver the interventions in a “crossed-therapist” design, with both therapists providing both treatments within the study.

### Study outcome measures

Along with a sociodemographic questionnaire, patients received the following questionnaires:

#### Primary outcome measure

- *Pelvic pain intensity* and *pelvic pain unpleasantness* were measured on NRS in a daily pain diary [39].

#### Secondary outcome measures

- *Endometriosis-related QoL* was measured by a validated Danish version of The Endometriosis Health Profile 30 questionnaire (EHP-30) [40, 41].
- *Workability* was measured by The Work Ability Index (WAI) [42, 43]. We used a linguistically validated Danish version.
- *Endometriosis-related symptoms* [4] were measured on NRS (from 0 = no symptom to 10 = worst symptom imaginable) in a weekly symptom diary [29].
- *Acceptance of chronic pain* was measured by a validated Danish version of The Chronic Pain Acceptance Questionnaire (CPAQ) [44–46].
- *Vaginal pressure pain detection threshold* (PPDT) was examined with a modified pressure algometer (palpometer) applicable for intravaginal pelvic floor muscle examination (FSR151, Interlink Electronics, Inc.). Due to large variability when measuring at other vaginal sites, the tissue around the sciatic spine was chosen for examination [30]. Participants were instructed to activate the pushbutton when pressure was perceived as pain. The average of six measurements (three on each side) was used to define PPDT.
- *Other measures*: At home, patients in the intervention groups filled out a daily home-work diary during the entire course of treatment.

### Sample size

Sample size was based on power analysis of a small randomized 3-armed pilot study (unpublished) for the primary outcome of pelvic pain between the groups: (1) PI vs. WL and (2) MY-ENDO vs. Non-specific and for the secondary outcome of QoL between the groups PI vs. WL. Pelvic pain was measured on NRS. The NRS scale score is standardized on a range from 0–10, defined by a mean of M = 6.0 and the standard deviation (SD) = 1.5. With the reasonable assumptions: Mean *n*1 = 6.0, Mean *n*2 = 5.0, SD = 1.5, power (1 − *β*) = 0.80, *α* = 0.05, two-sample, two-sided test, the number of participants needed would be 53 vs. 27 participants for differences between the groups PI vs. WL, and 36 vs. 36 participants for differences between the groups MY-ENDO vs. Non-specific. QoL was measured on EHP-30. The EHP-30 scale scores are standardized on a range from 0 to 100, defined by a mean of *M* = 50.0 and the standard deviation SD = 12.0. With the reasonable assumptions: Mean *n*1 = 50.0, Mean *n*2 = 40.0, SD = 12.0, power = 0.80, *α* = 0.05, two-sample, two-sided test, the number of participants needed would be 34 vs. 17 participants for differences between the groups PI vs. WL. Based on the power analyses it was planned to include 3 × 27 participants in the study.

### Statistical analysis

Baseline group differences were compared by the *χ*^2^-test or the Kruskal–Wallis test (due to non-normally distributed data). For continuous data, means and standard deviations were given. Normally distributed variables were compared using t-tests, and non-normally distributed variables were compared using non-parametric tests (i.e., Mann–Whitney). Study dropouts were defined as participants discontinuing the intervention or failing to return the questionnaires/diary. Mixed linear models (MLMs) were used to compare groups over time and to examine changes in outcomes over time within groups on all outcomes. MLMs tolerate missing values without compromising statistical power and take into account the nested nature of data. The MLM models were conducted using restricted maximum likelihood method (REML) and performed on an intention-to-treat basis. Data were hierarchically arranged with time as level one nested within individual as level two. Fixed effects were specified for intercept, time, group, and time × group interaction. All models included a random intercept, and a fixed slope was chosen due to the comparison of groups with small sample sizes. In order to compare end-point effects between measures, a linear function of time was estimated from baseline to post-treatment measure. All primary analyses were conducted blinded. Since analyses of the primary outcome yielded unexplained results, which were in contrast to the hypotheses, statistically significant (*P* < 0.05) baseline differences were entered as covariates in explorative post-hoc analyses of the primary outcome [47]. In addition, because of holiday periods, some patients completed an additional diary week, however, this holiday week was subtracted in the statistical analysis, and because some patients had missing values during the 12 weeks and some stopped completing the pain diary already after 11 weeks, sensitivity analysis was performed testing the robustness of the results. The sensitivity analyses were conducted using MLMs comparing the groups over time using last observation carried forward for missing values and comparing the groups over time after 11 weeks (week 12 was subtracted for all participants). Statistically significant results were defined as *P* < 0.05 (two-sided significance level). Effect sizes were expressed as Cohen´s *d,* with effect sizes of 0.2, 0.5, and 0.8 considered small, medium, and large, respectively. IBM SPSS statistics v.26 was used for all analyses.

## Results

### Patients

**Fig. 2:**
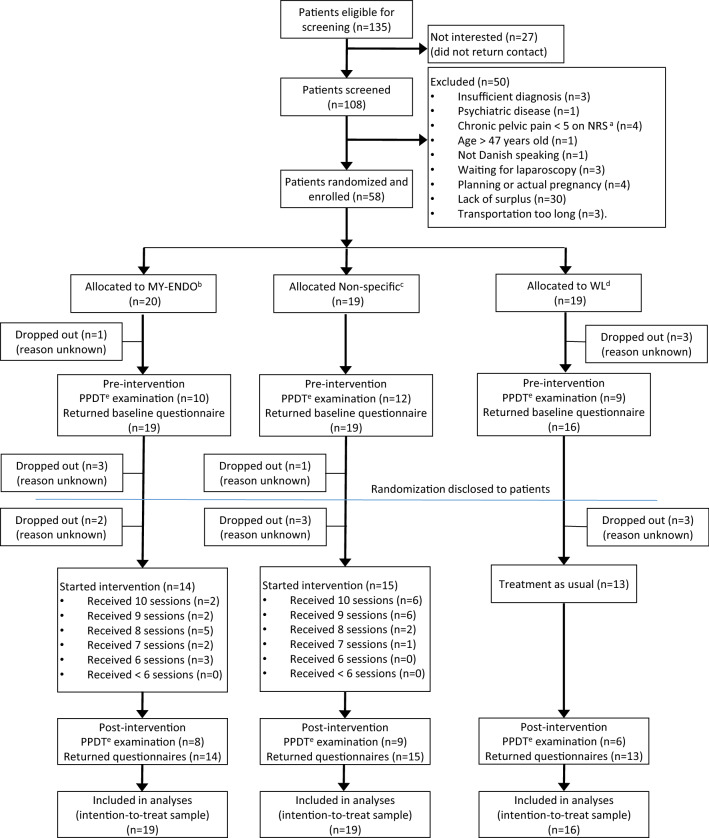
CONSORT study flow diagram

A total of 58 patients (*N*_MY-ENDO_ = 20, *N*_Non-specific_ = 19, *N*_WL_ = 19) were included in the study. The CONSORT study flow diagram is shown in Fig. 2. Four patients dropped out before baseline measurement was obtained. Another 12 patients dropped out before study completion. Reasons for dropout are unknown. There were no statistically significant differences in dropout rate between the groups (*P* = 0.856). Nor were there any statistically significant differences regarding baseline characteristics for dropouts (*N* = 12) compared to completers (*N* = 42) except for previous use of alternative treatments (*P* = 0.020) as none of the dropouts had any previous experience with alternative treatments for endometriosis compared to 33.3% of completers.

Sociodemographic, clinical, and pain-related data are shown in Table 2. A significant baseline difference was found between the three study arms for the use of pain medication (*P* = 0.050), and the use of pain medication was therefore adjusted for in post-hoc analyses of the primary outcome [47].

**Table 2:** Sociodemographic, clinical and pain-related baseline characteristics of the study sample

	MY-ENDO^a^ *N* = 19	Non-specific^b^ *N* = 19	WL^c^ *N* = 16
	Mean (SD)/%	Mean (SD)/%	Mean (SD)/%
Age	28.95 (7.84)	33.84 (7.69)	32.81 (9.01)
Marital status			
Married/living together Single Other	57.9% 36.8% 5.3%	52.6% 26.3% 21.1%	75.0% 18.8% 6.3%
Biological children			
0 1 2 3	78.9% 5.3% 15.8% 0.0%	57.9% 26.3% 5.3% 10.5%	56.3% 18.8% 18.8% 6.3%
Occupation			
Full time or more Part time Flexi job/rehabilitation Off work sick Unemployed Enrolled in education Other	31.6% 10.5% 10.5% 10.5% 5.3% 31.6% 0.0%	47.4% 15.8% 5.3% 10.5% 5.3% 10.5% 5.3%	25.0% 25.0% 6.3% 12.5% 0.0% 12.5% 18.8%
Weekly working hours	29.15 (15.90)	29.21 (14.62)	25.69 (14.87)
Level of vocational education			
No education Skilled Higher education < 3 years Higher education 3–4 years Higher education > 4 years Other	31.6% 0.0% 21.1% 26.3% 15.8% 5.3%	21.1% 0.0% 15.8% 52.6% 10.5% 0.0%	12.5% 6.3% 18.8% 25.0% 12.5% 18.8%
Years since diagnosis	5.42 (5.80)	7.21 (6.05)	7.06 (5.93)
Years since onset of pelvic pain	14.00 (8.28)	18.16 (6.01)	12.93 (7.51)
Year from symptom onset till diagnosis	7.47 (5.91)	9.61 (6.89)	5.07 (5.74)
Natural menopause	0	0	0
Number of endometriosis operations till today	1.68 (1.16)	2.84 (2.54)	3.13 (2.97)
Previous endometriosis treatment			
Removal of endometriosis lesions Hormonal treatment Pain medication Physical treatment Psychological treatment Alternative treatment	89.5% 100% 89.5% 57.9% 5.3% 26.3%	94.7% 100% 100% 31.6% 10.5% 36.8%	81.3% 87.5% 100% 50.0% 6.3% 12.5%
Current endometriosis treatment			
No treatment Hormonal treatment Pain medication	5.3% 84.2% 63.2%	0.0% 89.5% 94.7%	12.5% 75.0% 81.3%
Symptoms in the last week (NRS)^d^			
Pelvic pain	6.11 (2.05)	5.53 (1.90)	6.00 (1.51)
Dysuria Dyschezia Dysmenorrhea^e^ Dyspareunia^e^ Fatigue Constipation Diarrhea Nausea Vomiting	1.11 (1.97) 3.79 (2.68) 7.50 (1.60) 5.89 (2.15) 7.53 (1.71) 4.26 (2.86) 1.58 (2.91) 3.95 (2.46) 0.68 (1.77)	1.89 (2.51) 3.63 (2.06) 8.50 (0.71) 3.43 (2.23) 6.58 (2.43) 3.37 (2.73) 0.95 (1.61) 2.67 (2.14) 0.21 (0.63)	2.44 (2.50) 4.06 (2.84) 6.25 (4.19) 3.29 (3.15) 7.00 (1.90) 3.38 (3.44) 1.56 (2.56) 3.13 (3.12) 0.13 (0.50)

^a^Mindfulness- and Acceptance-based Psychological Intervention

^b^Matched non-specific psychological intervention

^c^Wait-list

^d^Numeric Rating Scale (0–10, 0 = no symptom, 10 = worst imaginable symptom)

^e^Group 1: dysmenorrhea *N* = 4, dyspareunia *N* = 7. Group 2: dysmenorrhea *N* = 2, dyspareunia *N* = 7. Group 3: dysmenorrhea *N* = 8, dyspareunia *N* = 9

### Psychological intervention vs. waitlist

Statistically significant time × group effects were found for pelvic pain intensity (*P* = 0.009, *d* = 0.23) and unpleasantness (*P* = 0.009, *d* = 0.22) (Table 3), due to pain reduction in the waitlist group. Comparable results were found in sensitivity analyses. However, when adjusting for use of pain medication in time × group interactions of the primary outcomes pelvic pain unpleasantness (*P* = 0.071, *d* = 0.59) no longer reached statistical significance (Table 3).

**Table 3:** Primary and secondary outcomes and estimates of treatment effects for PI vs. WL and MY-ENDO vs. non-specific

Time × group Interaction														
Outcomes	Psychological intervention (PI)		Waitlist (WL)		PI vs. WL			MY–ENDO^d^		Non–specific^e^		MY–ENDO vs. non–specific		
	Mean (SD)^a^ T1^b^	Mean (SD)^a^ T2^c^	Mean (SD)^a^ T1^b^	Mean (SD)^a^ T2^c^	Cohen´s *d*	*F*	*P*	Mean (SD)^a^ T1^b^	Mean (SD)^a^ T2^c^	Mean (SD)^a^ T1^b^	Mean (SD)^a^ T2^c^	Cohen´s *d*	*F*	*P*
**Primary outcomes**														
*Chronic pelvic pain*														
Pain intensity (NRS)	–	–	–	–	0.23	6.96	**0.009**	–	–	–	–	0.22	4.38	**0.037**
Pain intensity (NRS)^f^	–	–	–	–	0.66	4.22	**0.047**	–	–	–	–	0.59	2.27	0.144
Pain unpleasantness (NRS)	–	–	–	–	0.22	6.85	**0.009**	–	–	–	–	0.20	3.91	**0.049**
Pain unpleasantness (NRS)^f^	–	–	–	–	0.59	3.44	0.071	–	–	–	–	0.43	1.17	0.289
**Secondary outcomes**														
* EHP-30/Quality of Life*														
Pain	49.60 (17.51)	42.61 (19.77)	47.20 (19.99)	48.78 (12.61)	0.44	1.87	0.180	51.95 (20.60)	42.31 (21.87)	47.42 (14.45)	42.88 (18.54)	0.22	0.30	0.586
Control and powerlessness	65.37 (16.14)	48.21 (18.30)	60.26 (25.15)	60.26 (20.53)	0.78	6.03	**0.019**	68.15 (17.27)	50.00 (19.17)	62.78 (15.14)	46.67 (18.04)	0.11	0.07	0.801
Emotional wellbeing	44.97 (13.38)	33.62 (11.51)	47.44 (22.79)	48.71 (22.66)	1.01	10.17	**0.003**	47.32 (10.92)	39.29 (11.52)	42.78 (15.39)	28.33 (8.94)	0.55	2.04	0.165
Social support	50.00 (26.52)	37.07 (21.52)	53.85 (24.94)	53.37 (27.79)	0.66	4.41	**0.042**	51.34 (26.08)	43.75 (21.09)	48.75 (27.77)	30.83 (20.66)	0.59	2.36	0.136
Self-image	53.73 (26.41)	44.54 (24.43)	49.36 (23.93)	48.07 (30.08)	0.32	1.05	0.312	51.79 (29.63)	40.48 (28.09)	55.56 (23.92)	48.33 (20.70)	0.19	0.24	0.631
*Work Ability Index*	34.10 (8.49)	36.40 (7.84)	33.09 (7.94)	35.75 (5.29)	0.37	0.83	0.371	32.70 (9.95)	36.94 (8.80)	35.36 (7.16)	35.95 (7.37)	0.68	1.72	0.210
* Endometriosis-related symptoms (NRS)*														
Pelvic pain (total)	–	–	–	–	0.24	1.81	0.182	–	–	–	–	0.24	1.24	0.269
Dysuria	–	–	–	–	0.04	0.04	0.837	–	–	–	–	0.18	0.54	0.464
Dyschezia	–	–	–	–	0.43	4.05	**0.047**	–	–	–	–	0.20	0.79	0.378
Dysmenorrhea	–	–	–	–	0.05	0.07	0.786	–	–	–	–	0.11	0.77	0.381
Dyspareunia	–	–	–	–	0.06	0.09	0.760	–	–	–	–	0.05	0.03	0.868
Fatigue	–	–	–	–	0.13	0.46	0.501	–	–	–	–	0.11	0.29	0.593
Constipation	–	–	–	–	0.47	4.16	**0.045**	–	–	–	–	0.16	0.40	0.528
Diarrhea	–	–	–	–	0.18	0.90	0.344	–	–	–	–	0.25	0.03	**0.035**
Nausea	–	–	–	–	0.47	6.02	**0.016**	–	–	–	–	0.16	0.59	0.444
Vomiting	–	–	–	–	0.04	0.18	0.673	–	–	–	–	0.15	1.75	0.187
* Pressure pain detection threshold (PPT)*^g^	34.62 (12.12)	34.04 (15.81)	57.73 (41.31)	53.79 (25.77)	0.25	0.35	0.562	38.64 (16.75)	36.61 (21.60)	31.05 (4.20)	31.48 (7.36)	0.28	0.27	0.615
*Pain acceptance (total score)*	52.79 (15.92)	65.26 (17.26)	57.42 (21.90)	62.54 (22.71)	0.41	1.63	0.210	52.15 (19.35)	65.86 (20.66)	53.33 (12.92)	64.62 (13.50)	0.21	0.27	0.606
Activity engagement	34.31 (11.55)	40.62 (11.94)	35.17 (12.83)	37.62 (12.53)	0.44	1.85	0.182	35.36 (13.62)	42.07 (13.54)	33.33 (9.62)	39.08 (10.27)	0.02	< 0.01	0.966
Pain willingness	18.11 (8.47)	24.63 (7.86)	22.25 (9.91)	24.92 (11.47)	0.36	1.22	0.276	15.92 (9.23)	23.79 (10.01)	20.00 (7.56)	25.54 (4.86)	0.38	0.89	0.355

Statistically significant results (*P* < 0.05) are shown in boldface

^a^SD = Standard deviation, Mean and SD were calculated for outcomes measured at pre- and post-intervention, respectively. Chronic pelvic pain was measured daily in 84 successive days and endometriosis-related symptoms was measured weekly in 12 successive weeks (See also Table 4)

^b^T1 = Pre-intervention

^c^T2 = Post-intervention

^d^ Mindfulness- and Acceptance-based Psychological Intervention

^e^Non-specific psychological intervention

^f^Post-hoc analysis including the covariate “taking pain medication”

^g^*N*_MY-ENDO_ = 8, *N*_Non-specific_ = 8, *N*_WL_ = 6

We also found statistically significant time × group interactions for the QoL-subscales ‘control and powerlessness’ (*P* = 0.019, *d* = 0.78), ‘emotional wellbeing’ (*P* = 0.003, *d* = 1.01) and ‘social support’ (*P* = 0.042, *d* = 0.66), and for the endometriosis-related symptoms ‘dyschezia’ (*P* = 0.047, *d* = 0.43), ‘constipation’ (*P* = 0.045, *d* = 0.47) and ‘nausea’ (*P* = 0.016, *d* = 0.47) (Table 3).

### MY-ENDO vs. non-specific

Statistically significant time × group effects were found for pelvic pain intensity (*P* = 0.037, *d* = 0.22) and pelvic pain unpleasantness (*P* = 0.049, *d* = 0.20). The sensitivity analyses testing the robustness of the results yielded comparable results (Table 3). However, when adjusting for use of pain medication in time × group interactions of the primary outcomes neither pelvic pain intensity (*P* = 0.144, *d* = 0.59) nor pelvic pain unpleasantness (*P* = 0.289, *d* = 0.43) reached statistical significance (Table 3)*.*

Neither did we find any statistically significant time × group interactions for the secondary outcomes except for diarrhea (*P* = 0.035, *d* = 0.25) (Table 3).

With regards to the time spent on home practice, we did not find a significant difference between MY-ENDO (Mean = 22.41 min/day, SD = 20.93) and Non-specific (Mean = 22.26 min/day, SD = 15.47) in the average amount of time (min/day) spent on homework during the 10-week treatment period (*U* = 47.000, *N*_MY-ENDO_ = 10, *N*_Non-specific_ = 11, *P* = 0.605).

### Pre-post changes

Statistically significant pre-post changes for all three groups (MY-ENDO, Non-specific, and WL) are found in Table 4.

**Table 4:** Pre-post effects for all outcomes within the three groups MY-ENDO, Non-specific, and waitlist

Outcomes	Measurements (*N*)	MY-ENDO^a^ *N* = 14				Non-specific^b^ *N* = 15				WL^c^ *N* = 13			
		Time^e^	Cohen´s *d*	*P*	Estimate^f^ (SE)^g^	Time^e^	Cohen´s *d*	*P*	Estimate^f^ (SE)^g^	Time^e^	Cohen´s *d*	*P*	Estimate^f^ (SE)^g^
**Primary outcome**													
*Pelvic pain*													
Pain intensity (NRS)^d^	84	Linear	0.17	0.210	0.004 (0.003)	Quadratic	0.26	0.097	− 0.005 (0.003)	Linear	0.52	**0.001**	− 0.011 (0.003)
Pain unpleasantness (NRS)^d^	84	Linear	0.05	0.713	0.001 (0.003)	Quadratic	0.37	**0.016**	− 0.007 (0.003)	Log	0.60	** < 0.001**	− 0.014 (0.004)
**Secondary outcomes**													
*EHP-30/Quality of Life*^h^													
Pain	2	Linear	0.80	0.188	− 8.734 (6.254)	Linear	0.55	0.322	− 4.545 (4.425)	Linear	0.30	0.618	1.573 (3.071)
Control and powerlessness	2	Linear	1.75	**0.008**	− 18.09 (5.751)	Linear	1.63	**0.009**	− 16.11 (5.300)	Linear	0.00	1.000	< 0.001 (5.968)
Emotional wellbeing	2	Linear	1.61	**0.013**	− 8.036 (2.778)	Linear	2.23	**0.001**	− 14.44 (3.471)	Linear	0.25	0.677	1.282 (2.998)
Social support	2	Linear	1.05	0.080	− 7.589 (3.995)	Linear	1.80	**0.005**	− 17.92 (5.313)	Linear	0.06	0.915	− 0.481 (4.389)
Self-image	2	Linear	1.02	0.089	− 11.31 (6.153)	Linear	0.67	0.229	− 7.222 (5.742)	Linear	0.11	0.856	− 1.282 (6.924)
*Work Ability Index*	2	Linear	2.47	**0.021**	2.780 (0.905)	Linear	0.00	0.986	0.039 (2.065)	Linear	1.38	0.082	3.113 (1.573)
*Endometriosis-related symptoms (NRS)*													
Pelvic pain	12	Quadratic	0.00	0.992	< 0.001 (0.035)	Quadratic	0.49	0.143	− 0.061 (0.041)	Log	0.75	**0.037**	− 0.092 (0.042)
Dysuria	12	Log	0.15	0.704	− 0.012 (0.031)	Linear	0.38	0.241	− 0.038 (0.032)	Quadratic	0.39	0.372	− 0.041 (0.046)
Dyschezia	12	Linear	0.62	0.068	− 0.069 (0.036)	Exponential	1.02	**0.002**	− 0.107 (0.032)	Log	0.21	0.675	0.027 (0.062)
Dysmenorrhea	12	Linear	0.01	0.970	− 0.003 (0.080)	Linear	0.27	0.121	− 0.086 (0.055)	Linear	0.23	0.242	− 0.071 (0.060)
Dyspareunia	12	Linear	0.20	0.283	− 0.074 (0.069)	Linear	0.28	0.121	− 0.059 (0.038)	Linear	0.38	0.059	− 0.086 (0.045)
Fatigue	12	Log	0.40	**0.017**	− 0.075 (0.031)	Log	0.25	0.125	− 0.046 (0.030)	Log	0.19	0.282	− 0.033 (0.031)
Constipation	12	Linear	0.26	0.434	− 0.04 (0.048)	Cubic	0.67	0.080	− 0.076 (0.042)	Log	0.77	0.277	0.090 (0.078)
Diarrhea	12	Log	0.12	0.479	0.023 (0.032)	Linear	0.37	**0.026**	− 0.065 (0.029)	Linear	0.44	**0.014**	− 0.061 (0.024)
Nausea	12	Linear	0.09	0.573	0.019 (0.034)	Linear	0.13	0.434	− 0.025 (0.033)	Log	0.62	**0.001**	− 0.142 (0.040)
Vomiting	12	Log	0.24	0.148	− 0.045 (0.031)	Log	0.06	0.737	0.009 (0.027)	Log	0.50	**0.007**	− 0.030 (0.011)
*Pressure pain detection threshold (PPT)*^i^	2	Linear	0.39	0.621	− 2.031 (3.927)	Linear	0.11	0.888	0.280 (1.927)	Linear	0.26	0.734	− 5.902 (16.703)
*Pain acceptance (total score)*	2	Linear	2.28	**0.002**	13.82 (3.461)	Linear	2.11	**0.002**	11.40 (2.989)	Linear	0.51	0.401	5.558 (6.374)
Activity engagement	2	Linear	2.34	**0.001**	6.714 (1.595)	Linear	1.78	**0.009**	6.475 (2.087)	Linear	0.55	0.373	2.806 (3.030)
Pain willingness	2	Linear	2.06	**0.003**	7.748 (2.126)	Linear	1.50	**0.021**	5.227 (1.975)	Linear	0.45	0.454	2.776 (3.582)

^a^Mindfulness- and Acceptance-based Psychological Intervention

^b^Non-specific psychological Intervention

^c^Wait-list

^d^Numeric Rating Scale

^e^Time = the best fit of time for the model

^f^Estimate indicates whether there is a negative or positive change in scores with time

^g^SE = Standard error

^h^For EHP-30 lower values indicate better QoL

^i^*N*_MY-ENDO_ = 8, *N*_Non-specific_ = 8, *N*_WL_ = 6. Statistically significant results (*P* < 0.05) are shown in boldface

### Post-hoc analyses

**Fig. 3:**
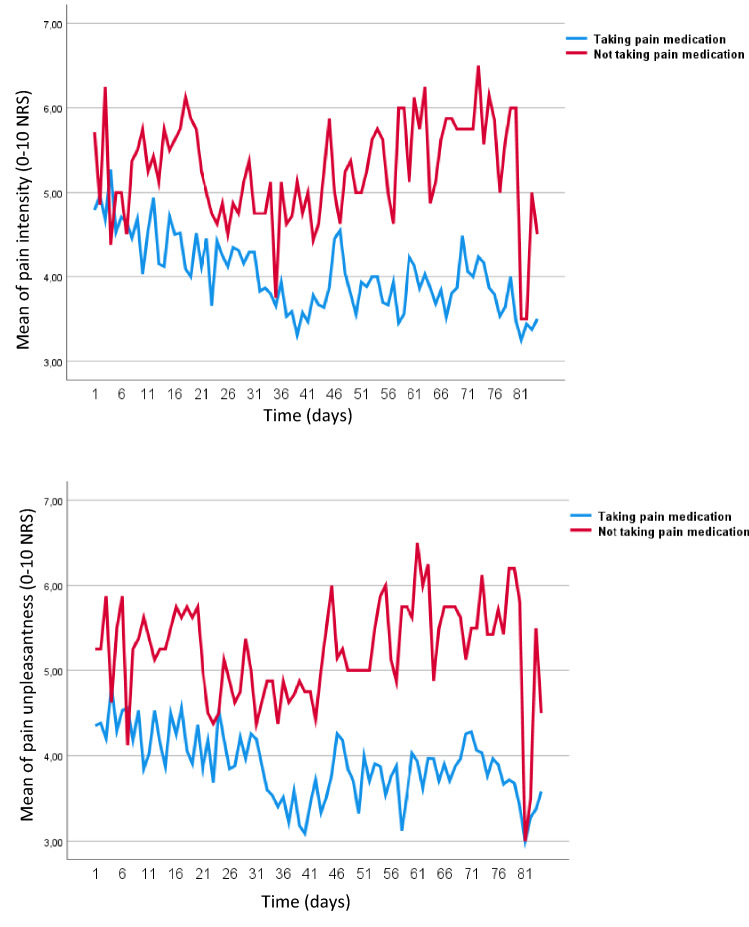
Differences in pelvic pain between the groups “taking pain medication” vs. “not taking pain medication”

When dividing participants into two groups (taking vs. not taking pain medication) independent of randomization allocation, statistically significant time × group effects were found for pelvic pain intensity (F = 11.3, *P* = 0.001, *d* = 0.29) and pelvic pain unpleasantness (F = 13.9, P < 0.001, *d* = 0.32). In the group taking pain medication, a significant reduction in pelvic pain intensity (*P* = 0.001, *d* = 0.32) and pelvic pain unpleasantness (P < 0.001, *d* = 0.44) was found, whereas in the group not taking pain medication, a significant increase in pelvic pain intensity (*P* = 0.039, *d* = 0.43) and pelvic pain unpleasantness (*P* = 0.048, *d* = 0.39) was found (Fig. 3).

### Therapist effects

Statistical analysis revealed no significant therapist x time interactions except for work ability (*P* = 0.021, *d* = 1.36) and nausea (*P* = 0.040, *d* = 0.24) and no statistically significant therapist x treatment interactions except for nausea (*P* = 0.029, *d* = 0.93).

There were no unprompted reports of any adverse events or side effects of the interventions. The results of MY-ENDO compared to WL and Non-specific compared to WL are found in Table 5.

**Table 5:** Primary and secondary outcomes and estimates of treatment effects for MY-ENDO vs. WL and Non-specific vs. WL

Outcomes	Time × group interaction					
	MY-ENDO^c^ vs. WL^d^			Non-specific^e^ vs. WL^d^		
	Cohen´s *d*	*F*	*P*	Cohen´s *d*	*F*	*P*
**Primary outcomes**						
*Chronic pelvic pain*						
Pain intensity (NRS)^ab^	0.42	1.08	0.310	0.71	3.16	0.087
Pain unpleasantness (NRS)^ab^	0.42	1.04	0.318	0.88	4.90	**0.036**
**Secondary outcomes**						
*EHP-30/Quality of Life*						
Pain	0.59	2.06	0.165	0.43	1.21	0.281
Control and powerlessness	0.87	4.73	**0.039**	0.79	4.10	0.053
Emotional wellbeing	0.91	5.21	**0.031**	1.32	11.39	**0.002**
Social support	0.48	1.44	0.241	0.97	6.16	**0.020**
Self-image	0.44	1.18	0.288	0.26	0.44	0.511
*Work Ability Index*	0.07	0.02	0.894	0.60	1.43	0.249
* Endometriosis-related symptoms (NRS)*						
Pelvic pain (total)	0.39	3.07	0.084	0.13	0.28	0.599
Dysuria	0.13	0.21	0.652	0.03	0.01	0.917
Dyschezia	0.37	1.72	0.196	0.56	4.09	**0.048**
Dysmenorrhea	0.08	0.40	0.529	0.05	0.05	0.828
Dyspareunia	0.04	0.02	0.884	0.06	0.20	0.656
Fatigue	0.19	0.68	0.413	0.10	0.17	0.686
Constipation	0.41	1.98	0.166	0.67	4.01	**0.050**
Diarrhea	0.40	2.87	0.095	0.02	0.03	0.858
Nausea	0.56	4.64	**0.035**	0.44	3.10	0.656
Vomiting	0.05	0.16	0.692	0.16	1.71	0.192
*Pressure Pain detection Threshold (PPT)*	0.17	0.10	0.755	0.22	0.18	0.678
*Pain acceptance (total score)*	0.47	1.30	0.265	0.34	0.72	0.405
Activity engagement	0.47	1.34	0.258	0.41	0.99	0.329
Pain willingness	0.49	1.46	0.239	0.25	0.39	0.539

Statistically significant results (*P* < 0.05) are shown in boldface

^a^Including “taking pain medication” as a covariate

^b^Numeric Rating Scale

^c^Mindfulness- and Acceptance-based Psychological Intervention

^d^Wait-list

^e^Non-specific Psychological Intervention

## Discussion

In this rigorous three-armed design we have demonstrated that psychological intervention (PI) does not significantly reduce pelvic pain in women suffering from endometriosis. Instead, PI led to large and significant improvements in QoL despite an ongoing experience of severe CPP.

Cochrane meta-analyses of psychological interventions have found that in a range of chronic pain conditions, lasting on average 9 years, Cognitive and Behavioral Therapy (CBT) shows small benefits in pain compared to active control conditions. Behavioral Therapy and ACT did not show such effects [22, 23]. The current study did not find pain reduction in the primary outcome which could be due to the specific therapy employed, the specific pain condition, and/or the fact that patients included in the study had suffered from chronic pelvic pain for more than 15 years on average and experienced significant pelvic pain (i.e., a daily NRS pain score ≥ 5). The results of the current study are in line with the general finding that it is difficult to demonstrate reduction in pain levels in well-controlled studies [22, 23]. Yet, despite these severe pain levels it was possible to significantly improve QoL.

The present study also found significant improvements in the endometriosis-related symptoms “dyschezia” and “constipation”. Studies indicate that bowel symptoms are frequent in endometriosis with interruptions in daily functioning [48]. These findings are therefore important and could be a result of patients in both groups starting to exercise as part of the intervention (e.g., yoga, mindful walking, and training exercises) since increased physical activity is found to be associated with decreased gastrointestinal symptoms in IBS [49] and may improve pain severity, physical function and QoL in other chronic pain patients [50].

Contrary to the majority of previous studies comparing mindfulness- and acceptance-based intervention to an active control [51–54], we did not find that MY-ENDO was superior to Non-specific on any outcomes. Importantly, it appears that a carefully matched non-specific control condition has not been used in previous studies [22, 23, 28, 51, 55], thereby underscoring the importance of using adequate control conditions [56]. The findings suggest that psychological interventions *in general* may be helpful in improving symptom management and QoL in patients suffering from endometrioses. This could potentially make psychological interventions more accessible for patients in clinical practice. Yet, future studies with larger sample sizes are needed to determine whether there might be specific benefits of adopting a mindfulness- and acceptance-based approach over a non-specific psychological approach in the management of endometriosis.

### Strengths and limitations

Notably, this study has some strengths that are rarely seen in RCTs examining the effects of psychological interventions. The inclusion of a carefully matched non-specific control condition and a no-treatment control in a 3-armed RCT together with the attempt to reduce significant threats to internal validity (e.g., balancing therapist training, adherence, and competence; controlling for therapist effects; ensuring an equal treatment structure across conditions; and balancing non-specific factors) entails that the findings of this study may be highly robust.

According to the power analysis we needed 81 participants to be able to detect significant differences in the primary outcome. Despite a smaller sample size, significant differences were found between the groups for the primary outcome *pelvic pain intensity* and *pelvic pain unpleasantness*. However, these differences appeared to be driven by significant improvements in the waitlist group and may be explained by differences in the use of pain medication. This explanation was further substantiated by the results of the post-hoc analysis and the results of the experimental pain test and it cannot be ruled out that the use of pain medication might have influenced the pain results.

While the small sample size would contribute considerably to the risk of type 2 error, one should also bear in mind that a large number of statistical tests were performed in the study, and hence there is considerable risk of type 1 error. Still, some interesting significant pre-post changes were found in our data including increased workability and lower fatigue in the MY-ENDO group. However, these effects did not show statistically significant differences between the groups, and larger sample sizes are needed in future studies to answer questions about specificity.

Recruiting participants was difficult due to patients suffering from physical disabilities and lack of energy making the 3-h in-person commitment a barrier to participation. Other barriers were geographical distance and working schedules.

Future studies could try new ways to overcome these limitations for example by stratifying participants by use of pain medication and offering digitally delivered therapy to this patient group.

### Conclusions

Standard treatment for endometriosis is primarily focused on hormonal treatment, pain medication, and surgery. However, medical treatment can induce serious side effects leading to discontinuation of treatment and recurrence of symptoms, and surgery that resolves endometriosis may not necessarily resolve pain since the extent of pain may be unrelated to the extent of disease [17].

With this rigorous three-armed RCT we have demonstrated that PIs specifically targeting endometriosis can lead to significant and large improvements in QoL and improvements in dyschezia and constipation despite an ongoing experience of severe CPP. Therefore, PIs aimed at symptom management and the improvement of QoL could be an appropriate supplement to an interdisciplinary endometriosis treatment.

## Data Availability

Data from this study is not publicly available due to restrictions pertaining to the general data protection regulation in Denmark but will be shared on reasonable request to the corresponding author.

## References

[1] Zondervan KT, Becker CM, Missmer SA (2020). Endometriosis. New England Journal of Medicine.

[2] Ferrero S, Arena E, Morando A, Remorgida V (2010). Prevalence of newly diagnosed endometriosis in women attending the general practitioner. International journal of gynaecology and obstetrics: The official organ of the International Federation of Gynaecology and Obstetrics.

[3] Seaman H, Ballard K, Wright J, De Vries C (2008). Endometriosis and its coexistence with irritable bowel syndrome and pelvic inflammatory disease: Findings from a national case–control study—Part 2. BJOG: An International Journal of Obstetrics & Gynaecology.

[4] Hansen KE, Kesmodel US, Baldursson EB, Kold M, Forman A (2014). Visceral syndrome in endometriosis patients. European Journal of Obstetrics, Gynecology, and Reproductive Biology.

[5] Schomacker ML, Hansen KE, Ramlau-Hansen CH, Forman A (2018). Is endometriosis associated with irritable bowel syndrome? A cross-sectional study. European Journal of Obstetrics & Gynecology and Reproductive Biology.

[6] Culley L, Law C, Hudson N, Denny E, Mitchell H, Baumgarten M, Raine-Fenning N (2013). The social and psychological impact of endometriosis on women’s lives: A critical narrative review. Human Reproduction Update.

[7] De Graaff AA, D'Hooghe TM, Dunselman GA, Dirksen CD, Hummelshoj L, Simoens S (2013). The significant effect of endometriosis on physical, mental and social wellbeing: Results from an international cross-sectional survey. Human Reproduction.

[8] Ferreira ALL, Bessa MMM, Drezett J, de Abreu LC (2016). Quality of life of the woman carrier of endometriosis: Systematized review. Reprodução & Climatério.

[9] Soliman AM, Coyne KS, Zaiser E, Castelli-Haley J, Fuldeore MJ (2017). The burden of endometriosis symptoms on health-related quality of life in women in the United States: A cross-sectional study. Journal of Psychosomatic Obstetrics & Gynecology.

[10] Marinho MCP, Magalhaes TF, Fernandes LFC, Augusto KL, Brilhante AVM, Bezerra L (2018). Quality of life in women with endometriosis: An integrative review. Journal of Women’s Health.

[11] Facchin F, Barbara G, Saita E, Mosconi P, Roberto A, Fedele L, Vercellini P (2015). Impact of endometriosis on quality of life and mental health: Pelvic pain makes the difference. Journal of Psychosomatic Obstetrics and Gynecology.

[12] Chen LC, Hsu JW, Huang KL, Bai YM, Su TP, Li CT, Yang AC, Chang WH, Chen TJ, Tsai SJ, Chen MH (2016). Risk of developing major depression and anxiety disorders among women with endometriosis: A longitudinal follow-up study. Journal of Affective Disorders.

[13] Gambadauro P, Carli V, Hadlaczky G (2019). Depressive symptoms among women with endometriosis: A systematic review and meta-analysis. American Journal of Obstetrics and Gynecology.

[14] Lorençatto C, Alberto Petta C, José Navarro M, Bahamondes L, Matos A (2006). Depression in women with endometriosis with and without chronic pelvic pain. Acta Obstetricia et Gynecologica Scandinavica.

[15] Jia S-z, Leng J-h, Shi J-h, Sun P-r, Lang J-h (2012). Health-related quality of life in women with endometriosis: A systematic review. Journal of Ovarian Research.

[16] Porpora MG, Pallante D, Ferro A, Crisafi B, Bellati F, Benedetti PP (2010). Pain and ovarian endometrioma recurrence after laparoscopic treatment of endometriosis: A long-term prospective study. Fertility and Sterility.

[17] Stratton P, Berkley KJ (2011). Chronic pelvic pain and endometriosis: Translational evidence of the relationship and implications. Human Reproduction Update.

[18] Brawn J, Morotti M, Zondervan KT, Becker CM, Vincent K (2014). Central changes associated with chronic pelvic pain and endometriosis. Human Reproduction Update.

[19] Soliman AM, Du EX, Yang H, Wu EQ, Haley JC (2017). Retreatment rates among endometriosis patients undergoing hysterectomy or laparoscopy. Journal of Women’s Health.

[20] Barra F, Grandi G, Tantari M, Scala C, Facchinetti F, Ferrero S (2019). A comprehensive review of hormonal and biological therapies for endometriosis: Latest developments. Expert Opinion on Biological Therapy.

[21] Leonardi M, Gibbons T, Armour M, Wang R, Glanville E, Hodgson R, Cave AE, Ong J, Tong YYF, Jacobson TZ, Mol BW, Johnson NP, Condous G (2020). When to Do surgery and when not to do surgery for endometriosis: A systematic review and meta-analysis. Journal of Minimally Invasive Gynecology.

[22] Williams ACC, Eccleston C, Morley S (2012). Psychological therapies for the management of chronic pain (excluding headache) in adults. Cochrane Database of Systematic Reviews.

[23] Williams ACC, Fisher E, Hearn L, Eccleston C (2020). Psychological therapies for the management of chronic pain (excluding headache) in adults. Cochrane Database of Systematic Reviews.

[24] Evans S, Fernandez S, Olive L, Payne LA, Mikocka-Walus A (2019). Psychological and mind-body interventions for endometriosis: A systematic review. Journal of Psychosomatic Research.

[25] Niekerk LV, Weaver-Pirie B, Matthewson M (2019). Psychological interventions for endometriosis-related symptoms: A systematic review with narrative data synthesis. Archives of Women’s Mental Health.

[26] Kold M, Hansen T, Vedsted-Hansen H, Forman A (2012). Mindfulness-based psychological intervention for coping with pain in endometriosis. Nordic Psychology.

[27] Hansen KE, Kesmodel US, Kold M, Forman A (2017). Long-term effects of mindfulness-based psychological intervention for coping with pain in endometriosis: A six-year follow-up on a pilot study. Nordic Psychology.

[28] Mohr DC, Ho J, Hart TL, Baron KG, Berendsen M, Beckner V, Cai X, Cuijpers P, Spring B, Kinsinger SW, Schroder KE, Duffecy J (2014). Control condition design and implementation features in controlled trials: A meta-analysis of trials evaluating psychotherapy for depression. Translational Behavioral Medicine.

[29] Bourdel N, Alves J, Pickering G, Ramilo I, Roman H, Canis M (2015). Systematic review of endometriosis pain assessment: How to choose a scale?. Human Reproduction Update.

[30] Brandsborg B, Dueholm M, Kehlet H, Jensen TS, Nikolajsen L (2011). Mechanosensitivity before and after hysterectomy: A prospective study on the prediction of acute and chronic postoperative pain. British Journal of Anaesthesia.

[31] Fields HL, Levine JD (1984). Placebo analgesia—a role for endorphins?. Trends in Neurosciences.

[32] Dunselman GA, Vermeulen N, Becker C, Calhaz-Jorge C, D’Hooghe T, De Bie B, Heikinheimo O, Horne AW, Kiesel L, Nap A, Prentice A, Saridogan E, Soriano D, Nelen W, European Society of Human Reproduction and Embryology (2014). ESHRE guideline: Management of women with endometriosis. Human Reproduction.

[33] Baer RA (2003). Mindfulness training as a clinical intervention: A conceptual and empirical review. Clinical Psychology: Science and Practice.

[34] Kabat-Zinn J (2013). Full catastrophe living: Using the wisdom of your body and mind to face stress, pain, and illness.

[35] Hayes SC, Luoma JB, Bond FW, Masuda A, Lillis J (2006). Acceptance and commitment therapy: Model, processes and outcomes. Behaviour Research and Therapy.

[36] Wampold BE, Mondin GW, Moody M, Stich F, Benson K, Ahn H-N (1997). A meta-analysis of outcome studies comparing bona fide psychotherapies: Empiricially, “all must have prizes”. Psychological Bulletin.

[37] Wampold BE, Imel ZE (2015). The great psychotherapy debate: The evidence for what makes psychotherapy work.

[38] Spielmans GI, Flückiger C (2018). Moderators in psychotherapy meta-analysis. Psychotherapy Research.

[39] Ferreira-Valente MA, Pais-Ribeiro JL, Jensen MP (2011). Validity of four pain intensity rating scales. Pain.

[40] Jones G, Kennedy S, Barnard A, Wong J, Jenkinson C (2001). Development of an endometriosis quality-of-life instrument: The endometriosis health profile-30. Obstetrics and Gynecology.

[41] Hansen KE, Lambek R, Røssaak K, Egekvist AG, Marschall H, Forman A, Kesmodel US (2021). Health-related quality of life in women with endometriosis: Psychometric validation of the endometriosis health profile 30 questionnaire using confirmatory factor analysis. Human Reproduction Open.

[42] de Zwart BCH (2002). Test-retest reliability of the Work Ability Index questionnaire. Occupational Medicine.

[43] van den Berg T, Elders L, de Zwart B, Burdorf A (2008). The effects of work-related and individual factors on the work ability index: A systematic review. Occupational and Environmental Medicine.

[44] McCracken LM, Vowles KE, Eccleston C (2004). Acceptance of chronic pain: Component analysis and a revised assessment method. Pain.

[45] Vowles KE, McCracken LM, McLeod C, Eccleston C (2008). The chronic pain acceptance questionnaire: Confirmatory factor analysis and identification of patient subgroups. Pain.

[46] la Cour P, Højsted J (2015). Validation of the Danish-language chronic pain acceptance questionnaire. Acta Anaesthesiologica Scandinavica.

[47] Hauck WW, Anderson S, Marcus SM (1998). Should we adjust for covariates in nonlinear regression analyses of randomized trials?. Controlled Clinical Trials.

[48] Hansen KE, Kesmodel US, Baldursson EB, Schultz R, Forman A (2013). The influence of endometriosis-related symptoms on work life and work ability: A study of Danish endometriosis patients in employment. European Journal of Obstetrics, Gynecology, and Reproductive Biology.

[49] Johannesson E, Simrén M, Strid H, Bajor A, Sadik R (2011). Physical activity improves symptoms in irritable bowel syndrome: A randomized controlled trial. American Journal of Gastroenterology.

[50] Geneen LJ, Moore RA, Clarke C, Martin D, Colvin LA, Smith BH (2017). Physical activity and exercise for chronic pain in adults: An overview of cochrane reviews. The Cochrane Library.

[51] Khoury B, Lecomte T, Fortin G, Masse M, Therien P, Bouchard V, Chapleau MA, Paquin K, Hofmann SG (2013). Mindfulness-based therapy: A comprehensive meta-analysis. Clinical Psychology Review.

[52] Bawa FL, Mercer SW, Atherton RJ, Clague F, Keen A, Scott NW, Bond CM (2015). Does mindfulness improve outcomes in patients with chronic pain? Systematic review and meta-analysis. British Journal of General Practice.

[53] Veehof MM, Trompetter HR, Bohlmeijer ET, Schreurs KMG (2016). Acceptance- and mindfulness-based interventions for the treatment of chronic pain: A meta-analytic review. Cognitive Behaviour Therapy.

[54] Hilton L, Hempel S, Ewing BA, Apaydin E, Xenakis L, Newberry S, Colaiaco B, Maher AR, Shanman RM, Sorbero ME, Maglione MA (2017). Mindfulness meditation for chronic pain: Systematic review and meta-analysis. Annals of Behavioral Medicine.

[55] Goyal M, Singh S, Sibinga EM, Gould NF, Rowland-Seymour A, Sharma R, Berger Z, Sleicher D, Maron DD, Shihab HM, Ranasinghe PD, Linn S, Saha S, Bass EB, Haythornthwaite JA (2014). Meditation programs for psychological stress and well-being: A systematic review and meta-analysis. JAMA Internal Medicine.

[56] Cohen SP, Vase L, Hooten WM (2021). Chronic pain: An update on burden, best practices, and new advances. The Lancet.

